# Title: insoluble proteins catch heterologous soluble proteins into inclusion bodies by intermolecular interaction of aggregating peptides

**DOI:** 10.1186/s12934-021-01524-3

**Published:** 2021-02-02

**Authors:** Jose Vicente Carratalá, Andrés Cisneros, Elijah Hellman, Antonio Villaverde, Neus Ferrer-Miralles

**Affiliations:** 1 Institute for Biotechnology and Biomedicine, Autonomous University of Barcelona, 08193 Bellaterra, Barcelona Spain; 2 Department of Genetics and Microbiology, Autonomous University of Barcelona, 08193 Bellaterra, Barcelona Spain; 3 Bioengineering, Biomaterials and Nanomedicine Networking Biomedical Research Centre (CIBER-BBN), 08193 Bellaterra, Barcelona Spain

**Keywords:** Recombinant protein, Inclusion body formation, Protein aggregation, Intermolecular interaction, Antimicrobial peptides

## Abstract

**Background:**

Protein aggregation is a biological event observed in expression systems in which the recombinant protein is produced under stressful conditions surpassing the homeostasis of the protein quality control system. In addition, protein aggregation is also related to conformational diseases in animals as transmissible prion diseases or non-transmissible neurodegenerative diseases including Alzheimer, Parkinson’s disease, amyloidosis and multiple system atrophy among others. At the molecular level, the presence of aggregation-prone domains in protein molecules act as seeding igniters to induce the accumulation of protein molecules in protease-resistant clusters by intermolecular interactions.

**Results:**

In this work we have studied the aggregating-prone performance of a small peptide (L6K2) with additional antimicrobial activity and we have elucidated the relevance of the accompanying scaffold protein to enhance the aggregating profile of the fusion protein. Furthermore, we demonstrated that the fusion of L6K2 to highly soluble recombinant proteins directs the protein to inclusion bodies (IBs) in *E. coli* through stereospecific interactions in the presence of an insoluble protein displaying the same aggregating-prone peptide (APP).

**Conclusions:**

These data suggest that the molecular bases of protein aggregation are related to the net balance of protein aggregation potential and not only to the presence of APPs. This is then presented as a generic platform to generate hybrid protein aggregates in microbial cell factories for biopharmaceutical and biotechnological applications.

## Background

Protein aggregation is an event widespread distributed from bacteria to animals. In bacteria, it has been related to stress states with the deployment of a complex protein network to compensate for the reduction of the ability of the cells to cope with the conformational stress [1]. In contrast, in yeast, protein aggregation is an inheritable adaptive phenomenon [2]. In animals, protein aggregation is also observed in pathological states related to conformational diseases [3], but is also associated to the formation of hormone aggregates in secretory granules [4].

During recombinant protein production, the detection of protein aggregates is a common outcome and is observed in both eukaryotic and prokaryotic expression systems [5, 6]. Bioinformatic tools are available for prediction of protein and peptide solubility and to identify aggregation-prone hot spots in the amino acid sequence, which can be modified during the design of recombinant genes [7–9]. However, changes in the primary structure of the natural proteins may lead to secondary effects as the appearance of immunogenic epitopes [10]. Therefore, the aggregation propensity of the produced protein may be reduced by lowering the transcription and translation rates of the gene, keeping intact the original amino acid sequence [11]. The main variables include the media composition, incubation temperature, promoter strength and inductor concentration among others [12].

In prokaryotes, the solubility of proteins is controlled by the protein quality control system, a complex network of protein factors involved in protein folding, unfolding and degradation [13]. In bacteria, aggregates, known as inclusion bodies (IBs), are dynamic protein clusters from which solubilized active protein conformers are released under physiological conditions [14–16]. In fact, recent experimental approaches have revealed the ability of active IBs to rescue enzymatic activities in cell cultures and to target cancer stem cells in cancer animal models [17–21]. Therefore, IBs are envisioned as depots of recombinant protein with the capacity to release the protein of interest from a complex and stable scaffold that protects the biological activity of the embed protein over time. Therefore, the enhancement of protein aggregation in this type of nanostructures is gaining interest. In fact, bioprocess design during recombinant protein production has been shown to impact the size of the IBs and the physicochemical quality of the recombinant protein achieving constant production of IBs [22–24]. In addition, aggregation propensity of recombinant proteins in expression systems may be enhanced by the addition of aggregation-prone peptides (APPs) in the design of the coding DNA sequence of the gene [25]. APP promote the establishment of intermolecular interactions between protein species enhancing the tendency of the resulting complexes to accumulate in the insoluble cell fraction [26]. Aggregation domains can be found in nature, in particular, several protein domains have been shown to possess such aggregation capacity, including a variant of the human β-amyloid peptide (Aβ42 (F19D)) [27], a mutant of the maltose binding protein (MalE31) [28], and the cellulose-binding domain of Clostridium cellulovorans (CBDclos) [29], among others. Another peptide with high capacity to enhance protein aggregation is VP1, a peptide sequence present in the VP1 structural protein of the *Foot-and-mouth disease virus* [26]. Due to safety and regulatory purposes, the use of viral protein domains, as VP1, is not suitable for some applications. For that reason, the development of novel APPs is of great interest.

In this study, we have selected a small APP of 8 amino acids in length (L6K2) to study the potential of this type of peptides to enhance the aggregation propensity of soluble proteins [30]. In addition, we have amplified its aggregating potential by protein engineering and demonstrated the antimicrobial activity of this type of amphipathic alpha-helices. We have also analyzed the role of stereospecific interactions in the aggregation of heterologous recombinant proteins in the presence of L6K2-containing peptides, providing a platform to obtain hybrid IBs inside the cells. These results have relevant implications in the biopharmaceutical and biotechnological applications of IBs.

## Results and discussion

### Modulation of recombinant protein solubility in *ClearCol*i cells

**Fig. 1:**
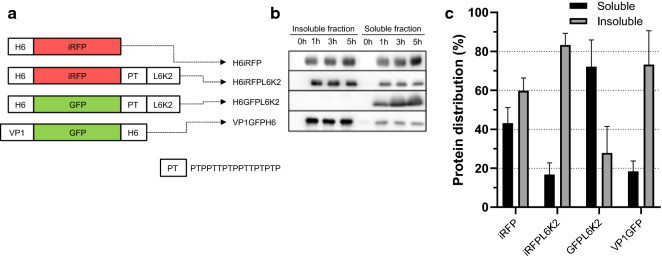
Effect of the addition of L6K2 in the aggregation propensity of fluorescent proteins. **a** Schematic representation of the recombinant genes is shown displaying protein domains by separate squares. H6 indicates the presence of a Hisx6 tag. PT corresponds to a linker with the indicated amino acid sequence. **b** Comparative aggregation propensity of iRFP and GFP in the presence of L6K2. VP1GFPH6 corresponds to a GFP fused to VP1 considered as positive control of the experiment. **c** Relative solubility (%) of recombinant proteins detailed in panel **a** analyzed by Western Blotting. Equivalent number of transformed *ClearColi* cells were lysed and soluble and insoluble cells fractions were separated. All proteins were detected with anti-his antibody

In order to study the performance of APP fused to recombinant proteins in the endotoxin free *ClearColi*™ expression system, two model soluble proteins; iRFP (near-infrared fluorescent protein) and GFP (green fluorescence protein) were selected as scaffolds and fused to the surfactant-like peptide L6K2, previously described as APP (Fig. 1a) [14].

In transformed *ClearColi* cells, recombinant H6iRFP protein was equally distributed in both soluble and insoluble cell fractions (Fig. 1b, c). As expected, upon L6K2 fusion, a different distribution pattern was observed, where most of the protein was located in the insoluble cell fraction, suggesting an increased aggregation tendency for this fusion protein (Fig. 1b, c). The change in solubility pattern was achieved within 1 h of induction and was maintained for up to 5 h (Fig. 1b). As a model APP, with high ability to enhance aggregation tendency of recombinant proteins, VP1 from the capsid protein of the *Foot-and-mouth disease virus* [26, 27] was fused to GFP (Fig. 1a). As expected, most of the protein signal in the sample was detected in the insoluble cell fraction (Fig. 1b, c). In contrast, the recombinant H6GFPL6K2 was mostly detected in the soluble cell fraction (Fig. 1b, c). These results indicated that the aggregation propensity of a recombinant protein may be modulated by APP although the solubility tendency of the scaffold protein may counteract this effect (compare solubility of iRFP and GFP when fused to L6K2 in Fig. 1b, c). In the case of VP1, the aggregation tendency overcome the high solubility of the GFP, while GFP solubility was not affected by the L6K2 addition (compare solubility of GFP when fused to VP1 or L6K2 in Fig. 1b, c).

### Impact of APP length on protein solubility in *ClearColi* cells

**Fig. 2:**
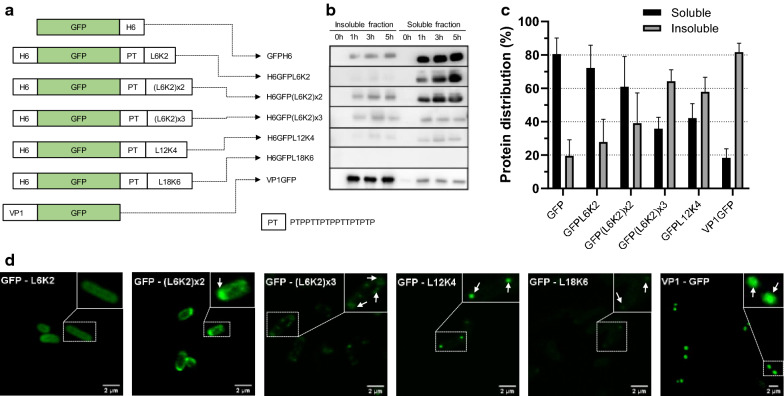
Production of GFP-containing recombinant proteins fused to aggregation prone peptides in *ClearColi*. **a** Schematic representation of L6K2-containing GFP constructs. H6 indicates the presence of a Hisx6 tag. PT corresponds to a linker with the indicated amino acid sequence. VP1 corresponds to the VP1 structural protein of *Foot-and-mouth disease virus* with high tendency to aggregate. **b** Detection of GFP in soluble and insoluble cell fractions of *ClearColi* transformed with expression plasmids containing the corresponding recombinant genes. **c** Relative solubility (%) of recombinant proteins detailed in panel **a** analized by Western Blotting. **d** Confocal analysis of *ClearColi* cultures producing GFP with several aggregation-prone peptides. Arrows indicate the distribution of protein aggregates in the expressing cells

As the ability of L6K2 to reduce solubility of GFP was not significant while it was effective in iRFP, we decided to evaluate the effect of peptide length in the solubility of GFP. For that, we redesigned the H6GFPL6K2 recombinant gene to add at the C-terminus of the GFP sequence, different versions of the surfactant-like peptide L6K2 (Fig. 2a). The aggregating potential of L6K2 peptide was amplified by reiteration of leucine and lysine repeats in different positions (see Table 1) and analyzed by AGGRESCAN software [8]. Selected peptides displayed higher hot spot area (HSA) than the original L6K2 peptide. However, only L12K4 and L18K6 showed increased normalized hot spot area (NHSA) and increased average aggregation-propensity hot spot (a^4^vAHS).

**Table 1:** Predictions of “hot spots (HS)” of aggregation in aggregating polypeptides by AGGRESCAN [8]

Name	HS region	HS size	Sequence	HSA	NHSA	a^4^vAHS	Ref.
L6K2	1–6	6	LLLLLLKK	6.211	1.035	0.949	[22]
(L6K2)x2	1–14	14	LLLLLLKKLLLLLLKK	12.789	0.913	0.865	This study
(L6K2)x3	1–22	22	LLLLLLKKLLLLLLKKLLLLLLKK	19.367	0.880	0.842	This study
L12K4	1–13	13	LLLLLLLLLLLLKKKK	14.625	1.125	1.074	This study
L18K6	1–19	19	LLLLLLLLLLLLLLLLLLKKKKKK	23.025	1.212	1.171	This study

*HS* hot spot, *HSA* hot spot area, *NHSA* normalized HSA, *a4vAHS* average aggregation-propensity in each HS

As previously observed, H6GFPL6K2 was detected in the soluble cell fraction of transformed *ClearColi* cells (Fig. 2b, c) and consequently, the emitted fluorescence was homogenously distributed in the cytosol (Fig. 2d). The addition of the L6K2 derived peptides had a positive impact in protein aggregation tendency. As expected, the distribution of fluorescence in the transformed cells was detected in protein clusters (IBs; Fig. 2d and Additional file 1: Fig S1a). In fact, we detected two different aggregation patterns in the L6K2 derived constructs. On the one hand, the proteins containing serial L6K2 repeats ((L6K2)x2 and (L6K2)x3) were preferentially detected in periplasmic areas around the cells, while the constructs containing longer Leucine/Lysine tracks (L12K4 and L18K6) were detected as fluorescent cellular pole aggregates. Therefore, the serial L6K2 repeats acted both as APP and periplasm localization signals since the fluorescence pattern revealed the clustering of signal in discrete aggregates on the periphery of the cell cytoplasm. In addition, the aggregation tendency in L6K2 repeats increased with the number of repeats while L12K4 presented an aggregation pattern like the observed in cells expressing the positive control VP1GFP. This aggregation tendency was not recorded in Western Blot analysis of the soluble and insoluble cell fractions (Fig. 2b, c), indicating that the aggregation tendency of the L6K2-derived peptides may be sensitive to the tested experimental conditions of protein extraction. This was not the case of the aggregation pattern of VP1GFP construct that was perfectly replicated under confocal laser scanning microscopy and SDS-PAGE (Compare VP1GFP data in Fig. 2b–d).

### Antimicrobial activity of L6K2-containing recombinant proteins

**Fig. 3:**
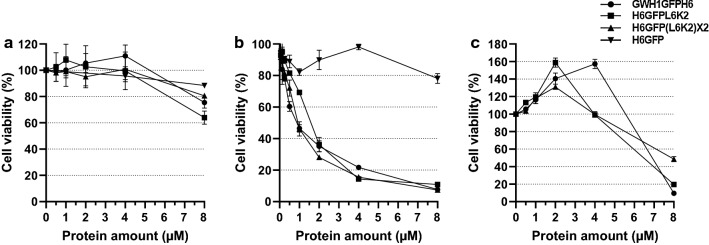
Survival curves of microbial cells in the presence of putative antimicrobial peptides fused to GFP protein. **a** *Staphylococcus aureus*, **b** *Escherichia coli* and **c.** *Micrococcus luteus*. The results are presented as mean of two replicas for each analyzed point with corresponding standard error bar. Similar CFU where seeded on 96-well plates and the indicated protein concentrations were added to evaluate its effect on cell metabolism

During recombinant gene expression experiments of L6K2-containing constructs, the growth of transformed *E. coli* cells was compromised, especially in the case of L18K6 (data not shown). At that point, we reasoned whether the peptides were toxic to the cell by displaying antimicrobial activity. The modeling of the L6K2 derived peptides with PEP-FOLD 3 [31–33] displayed amphipathic alpha helices in all cases (Additional file 2: Figure S2a). The preferred conformation of the L6K2-containing peptides was maintained in the presence of the PT linker, which has been described as a flexible peptide for separating protein domains (Additional file 2: Fig. S2b) [34]. This configuration has been described in naturally produced or synthetic cationic antimicrobial peptides (AMP) which have been proposed as a potential new class of antimicrobial drugs [35]. The production of small peptides is difficult to be reached by recombinant technologies due to reduced stability, and alternative strategies have been taken to overcome such a main bottleneck [36]. One possibility is the fusion of AMP to partner proteins for a potential dual effect on the final product. First, the reduction of the toxicity of the AMP over the expressing host, and the improvement in the stability of the peptide in expression systems [37]. However, the study of their activity when fused to reporter proteins by genetic engineering has not been explored in depth. Examples of this strategy include the fusion between GWH1 [38] and GFPH6 [14, 39] and the secretory production of AMP-containing fusion partners [40]. In those studies, the fusion of the AMP to the N-terminus of recombinant protein preserved the bactericidal activity of the AMP even though with its C-terminus anchored by the fusion. Therefore, we analyzed the putative antimicrobial activity of the purified soluble versions of H6GFPL6K2 and H6GFP(L6K2)X2 proteins and compared these data with that obtained for purified GWH1GFPH6. The results indicated that the antimicrobial activity of L6K2-containing recombinant proteins is strain specific (Fig. 3), being comparable to the antimicrobial activity of GWH1 peptide fused to GFP in *E. coli* cultures (Fig. 3b). In addition, the position of the peptide at each end of the scaffold protein did not appear to be relevant to the antimicrobial activity. On the other hand, the incubation of *S. aureus* with the proteins containing amphipathic alpha-helices had only a slight effect on cell viability under the tested conditions (Fig. 3a). Interestingly, the antimicrobial activity of the recombinant proteins was completely different when *Micrococcus luteus* cells were challenged. The addition of the purified proteins had a positive effect on cell viability at lower concentrations while at the highest protein concentration (8 µmol/L) the cell viability dropped drastically (Fig. 3c).

As observed in Fig. 3b, the antimicrobial activity of the L6K2-containing constructs was detected in *E. coli* cultures at low protein concentrations. This mechanism may explain the cell growth inhibition observed in *ClearColi* cultures transformed with expression vectors with cloned L6K2-derived genes. Small cationic or amphipathic molecules, similar to the ones described in this work, have been described as produced by prokaryotes and eukaryotic organisms as defense against infectious agents. These molecules belong to a non-specific ancient system of innate immunity and they perform their activity through direct interaction with membranes, nucleic acids, protein or even activate autolysins [41–44]. In the case of interacting with membranes, they cause the destabilization of the cytoplasmic membrane by forming pores or by their arrangement parallel to the membrane surface, disrupting the proton motive force and provoking the leakage of vital molecules which lead to cell death. However, even though their mechanism of action is nonspecific, it has been described a differential efficacy of the same antimicrobial peptide between Gram-negative and Gram-positive bacteria [45, 46]. In the case of Gram-positive bacteria, apart from membrane disruption, the reaction requires further interactions with the cell wall [45].

### Pull-down effect on aggregation tendency of H6GFPL6K2

**Fig. 4:**
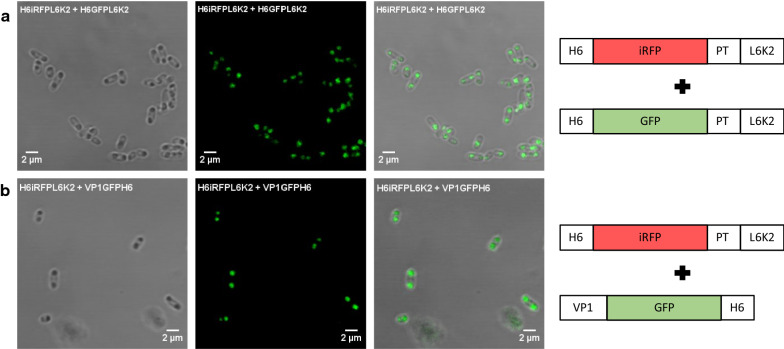
Confocal microscopy images of recombinant GFP in co-expression experiments. **a** Detection of H6GFPL6K2 in expressing H6iRFPL6K2 cells. **b** Detection of VP1GFPH6 in expressing H6iRFPL6K2 cells. A schematic representation of the corresponding constructs is depicted beside the confocal Microscopy images

Aggregation of different proteins may be enhanced by the stereospecific interaction of APP in bacteria [26]. In that context, we reasoned that the aggregation ability of a recombinant protein with the same APP may enhance the aggregation tendency of H6GFPL6K2 when produced simultaneously in cells. For that purpose, we generated a dual expression vector including the gene encoding for H6iRFPL6K2, which displayed a high tendency to aggregate beside the gene coding for H6GFPL6K2 to be simultaneously expressed. In cells expressing at the same time the aggregation prone H6iRFPL6K2 construct and the soluble H6GFPL6K2 construct, the fluorescence of the GFP shifted from the cytoplasm to polar protein aggregates (IBs) (Fig. 4). The green fluorescence distribution in expressing cells was similar to the pattern observed when co-expressing VP1GFPH6 and H6iRFPL6K2.

The change in the aggregation propensity of the H6GFPL6K2 seemed to be directed by the pull-down ability of the L6K2 peptide present in the H6iRFPL6K2 construct. The intermolecular interactions between L6K2 present in the two proteins enhances the aggregation tendency of GFP. In the expressing cells, the newly formed H6GFPL6K2, when interacting with H6iRFPL6K2 with a high tendency to aggregate was dragged to the insoluble cell fraction. Therefore, it may be hypothesized that when two different proteins share aggregation prone domains, even if one of the proteins is still soluble, the protein with the highly aggregation propensity may lead the accompanying soluble protein to the insoluble cell fraction through coexpression. However, although the secondary structure of the iRFP and GFP proteins is not similar (Additional file 3: Figure S3), the effect of the iRFP scaffold protein in the aggregation enhancement of H6GFPL6K2 may not be ruled out. For that reason, a spectral variant of GFP (EBFP2; highly similar in amino acid sequence and secondary structure) was fused to VP1 domain generating VP1EBFP2H6 construct (Additional file 4: Figure S4). Predictably, when produced recombinantly, this protein was mainly accumulated in the insoluble cell fraction (Additional file 5: Figure S5).

**Fig. 5:**
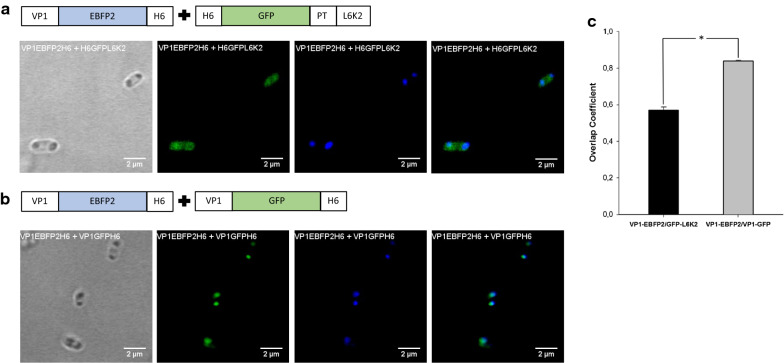
Overlay fluorescence images of H6GFPL6K2 and VP1GFPH6 with VP1EBFP2H6. **a** Detection of fluorescence emission from H6GFPL6K2 and VP1EBFP2H6 coexpressing cell cultures. **b** Detection of fluorescence emission from VP1GFPL6H6 and VP1EBFP2H6 coexpressing cell cultures. **c** Quantitative colocalization analysis of EBFP2 and GFP fluorescence signals in coexpression experiments. Overlap coefficients between the different fluorescences emitted by the fusion proteins VP1EBFP2H6/VP1GFPH6 and VP1EBFP2H6/H6GFPL6K2 expressed in *ClearColi* cells. Analysis performed from images obtained by confocal microscopy. *p < 0.001, one-way analysis of variance (ANOVA)

The distribution of the GFP fluorescence in cells simultaneously transformed with plasmids coding H6GFPL6K2 and VP1EBFP2H6 was homogeneously distributed in the cytoplasm of the cells, in agreement with the data obtained in the expression experiment of H6GFPL6K2 alone (compare the distribution of GFP fluorescence in Figs. 2c and 5a, and Additional file 1: Fig. S1b). On the other hand, the fluorescence emitted by EBFP2 fused to VP1 in those cells was mainly detected in polar IBs as expected. When VP1GFPH6 was expressed along with VP1EBFP2H6, the GFP fluorescence was located exclusively at the poles of the cells, as IBs (Fig. 5b and Additional file 1: Fig. S1b). The colocalization analysis of the fluorescence emission from both proteins indicated the preference of H6GFPL6K2 to aggregate in the presence of the same APP (Fig. 5c) ruling out an aggregating role of the scaffold protein in this process. Therefore, this result has a direct application for biopharmaceutical and biotechnological applications through protein engineering. In fact, these protein nanoclusters have been described as a source of soluble active protein obtained upon incubation in non-denaturing conditions [14–16] and have also been administered as biocompatible depots for tumor targeting of therapeutic proteins [17–21]. Furthermore, protein aggregation seems to be a common mechanism described in most of the expression systems [47–49] that opens up the possibility of expanding this type of strategy to proteins that are difficult to produce in prokaryotes. Therefore, the fusion of common APP to different therapeutic recombinant proteins can induce the colocalization of two recombinant proteins in IBs, obtaining protein formulations with potential synergic activities.

## Conclusions

Protein aggregation is a universal event which is associated to conformational diseases in eukaryotes. In bacteria, although it has been described as a symptom of metabolic stress resistance, some studies suggest the relevance of protein aggregation in physiological adaptation to stress [1]. In most of the recombinant protein production experiments described so far, a variable portion of the protein accumulated in bacterial IBs. In recent years, the use of IBs as active protein deposits has begun to be explored for biopharmaceutical and biotechnological applications [22, 50]. The current study highlighted the ability to enhance protein aggregation by the fusion of APPs to recombinant proteins used as baits for the capture of soluble proteins. This effect was even observed for highly soluble proteins as GFP. In addition, hybrid IBs, enriched in two different recombinant proteins, were formed through stereospecific interactions between common APP. Therefore, the presented data described the potential of APP in the control of the aggregation propensity of recombinant proteins in biological formulations based on IBs and open up the possible exploration of synergic activities of hybrid protein aggregates, produced in bacterial cell factories, for biomedical and nanobiotechnological purposes.

## Methods

### Molecular cloning

All protein designs were cloned in pETDuet™-1 plasmid (Novagen), except for H6iRFP, GFPH6 an GWH1GFPH6, which were cloned into *Nde*I and *Hind*III sites of plasmid pET22b (Novagen). For all pETDuet™-1 derived expression vectors, protein-coding DNA fragments were inserted in either, MCS1 or MCS2 of pETDuet™-1 plasmid. In the case of H6GFPL6K2, H6GFP(L6K2)X2, H6GFP(L6K2)X3, H6GFPL12K4, H6GFPL18K6 and VP1GFPH6, digestion was performed with *Nde*I and *Xho*I and insertion into the MCS2. On the other hand, H6iRFPL6K2 and VP1EBFP2H6 were digested with *Nco*I and *Hind*III and inserted into the MCS1. For dual expression plasmids, H6iRFPL6K2 + H6GFPL6K2, H6iRFPL6K2 + VP1GFPH6, VP1EBFP2H6 + VP1GFPH6 and VP1EBFP2H6 + H6GFPL6K2 a two-step cloning strategy was followed. After the generation of the MCS2 cloning plasmids (pETDuet-H6GFPL6K2 and pETDuet-VP1GFPH6), H6iRFPL6K2 and VP1EBFP2H6 fragments were inserted into the MCS1 after digestion with *Nco*I and *Hind*III. All L6K2-containing protein versions included a linker (PT) between GFP and the L6K2 peptide or derivatives as previously described [30].

### Expression of recombinant proteins in *ClearColi* cells

*ClearColi* BL21 (DE3) was selected as expression host for the different versions of the fluorescent proteins. The same conditions were applied in all cases. Briefly, after transformation with the corresponding expression vector, bacterial cells were allowed to growth in lysogenic broth (LB) medium supplemented with 100 µg/ml ampicillin in a shake flask (250 rpm) at 37 ºC. When cultures reached an optical density of approximately 0.5–0.6, protein expression was induced by adding 1 mmol/L isopropyl-β-D-thiogalactopyranoside (IPTG). Protein samples were taken at the indicated times (h) postinduction. In all cases, bacterial OD was measured and adjusted to 1, subsequently cells were collected by centrifugation (5 minutes, 1,200 *g*). Resuspended cells were processed for confocal microscopy visualization or to evaluate the relative protein distribution between the insoluble and soluble cell fractions, in those cases, the expression time was set at 3 h.

### Evaluation of protein aggregation propensity

Bacterial pellets harboring the expressed proteins were resuspended in 1 mL of PBS until a homogeneous suspension was achieved. Bacterial cell disruption was carried out by sonication (1 round of 1 min at 10 % amplitude and 1 round of 1 min at 15 % amplitude). Then, the soluble and insoluble cell fractions were separated by centrifugation (15 min, 15,000 g at 4 °C). The insoluble cell fraction, containing the cell debris, was resuspended in 1 mL of PBS, after that, a small aliquot of both fractions, soluble and insoluble, was mixed (1:1) with Laemmli buffer. Soluble samples were boiled at 90 ºC for 10 min, while the insoluble samples were boiled for 40 min. The processed samples were charged on SDS-PAGE gels and analyzed by Western Blotting with an anti-His monoclonal antibody (His Tag Antibody, mAb, Mouse, Genscript). Images were acquired with the ChemiDoc™ Touch Imaging System (Bio-Rad) and further processing was performed with Image Lab Software. Percentage of aggregation was calculated based on the numerical band intensity value obtained from blotting membrane images. For each expression time, the total amount of protein (100 %) was considered as the sum of the band intensities in both, soluble and insoluble cell fractions. Therefore, percentage of aggregation can be estimated from the band intensity value in the insoluble cell fraction.

### Visualization of recombinant proteins in *ClearColi* cells

Bacterial pellets harboring the expressed proteins were resuspended in 500 µL of PBS containing 4 % formaldehyde. Then, resuspended samples were incubated 10 minutes at RT and washed twice with PBS. In a glass slide, a small drop of ProLong™ Gold Antifade Mountant (Thermo) was mixed with 5 µL of the bacterial suspension. The resultant solution was covered with a coverslip and fixed to avoid dehydration. The observation of the fluorescent proteins inside bacteria was recorded by TCS-SP5 confocal laser scanning microscopy (Leica Microsystems). Images were processed using the ImageJ software. Colocalization analysis of fluorescent proteins in *ClearColi* cells were performed by measuring the overlap coefficients of 10 regions of interest (ROIs) which were compared by one-way analysis of variance (ANOVA).

### Purification of soluble recombinant proteins fused to APP

For purification of H6GFPL6K2, H6GFP(L6K2)X2 and GWH1GFPH6, protein expression was induced with 0.1 mmol/L isopropyl-β-D-thiogalactopyranoside (IPTG) at 20 ºC, overnight. The cell pellet was collected (6000 *g*, 4 ºC, 15 minutes) and resuspended in wash buffer (20 mmol/L Tris-HCl, pH 8.0, 500 mmol/L NaCl, 10 mmol/L imidazole) with ethylenediamine tetra-acetic acid-free protease-inhibitor (complete EDTA-Free, Roche). Cells were then disrupted by sonication (1 round of 2 min at 10 % amplitude and 10 rounds of 2 min at 15 % of amplitude) and cell debris was separated from soluble fraction by centrifugation (15,000 *g* at 4 ºC, 45 minutes). After filtration (0.22 µm), the His-tagged proteins were purified from the soluble fraction by His tag affinity chromatography using HiTrap Chelatin HP 1 ml column (GE Healthcare) in an ÄKTA purifier FPLC (GE Healthcare). The purified fraction was obtained after elution with a linear gradient of 20 mmol/L Tris-HCl pH 8.0, 500 mmol/L NaCl, 500 mmol/L imidazole. The purity of the different samples was analyzed by TGX gel chemistry and Western Blotting. The selected fractions were mixed and dialyzed against sodium bicarbonate buffer with salt (166 mmol/L NaHCO3, pH 8.0, 333 mmol/L NaCl) and protein amounts were quantified by Bradford assay.

### Antimicrobial activity of APP-containing recombinant proteins

The antimicrobial activity of H6GFPL6K2, H6GFP(L6K2)X2 and GWH1GFPH6 was evaluated against three bacterial species, *E. coli*, *S. aureus*, and *M. luteus*, using the broth micro-dilution method. Different two-fold dilutions of the proteins, ranging from 0.06 to 8 µmol/L, were seeded in 96-well plates for each bacterial species. After that, 10^6^ CFU/mL of the corresponding bacteria were inoculated in each well. Maximal growth was achieved in control wells with no protein. Each concentration was evaluated in technical duplicates. Wells with 100 µL of Mueller Hinton Broth Cation-adjusted medium (MHB-2, Sigma-Aldrich) were considered as blank solution. Growth conditions were stablished in 18 h at 37 ºC. The bacterial viability was evaluated using the commercially available BacTiter-Glo™ Microbial Cell Viability Assay (Promega) following the manufacturer’s instructions. Luminescence was measured using the Multilabel Plater Reader VICTOR3 (PerkinElmer).

## Supplementary Information

**Additional file 1: Figure S1.** Extended confocal microscopy visualization of *E. coli ClearColi* cells producing the recombinant fluorescent proteins. **a** Visual field of cells producing L6K2-containing constructs and VP1GFPH6 (aggregation control) analyzed in Fig. 2. **b** Visual fields of cells producing simultaneously GFP and EBFP2 constructs analyzed in Fig 5. Scale bar indicates 4 μm.

**Additional file 2: Figure S2.** PEP-FOLD server-generated models of aggregation prone peptides fused to GFP scaffold protein. **a** Regular polypeptide helices in a right-handed alpha-helical conformation are shown. All structures are reproduced at the same scale. **b** Helical conformation of two L6K2-containing peptides (blue) in the presence of PT linker (grey).

**Additional file 3: Figure S3.** DNA sequences of recombinant genes H6iRFPL6K2 and H6GFPL6K2 used in the study. Translate tool from Expasy was used to obtain corresponding amino acid sequences. Clustalw was run to align amino acid sequences and Swiss Model to display 3D structures of the recombinant proteins.

**Additional file 4: Figure S4.** DNA sequence of recombinant gene VP1EBFP2H6 used in the study. Translate tool from Expasy was used to obtain corresponding amino acid sequence. Clustalw was run to align amino acid sequence of GFP and EBFP2. Swiss Model was used to display 3D structure of EBFP2 protein.

**Additional file 5: Figure S5.** Relative solubility (%) of recombinant proteins. Detection of H6GFPL6K2, VP1GFPH6 and VP1EBFP2H6 in the soluble and insoluble cell fractions of *ClearColi* analyzed by Western Blotting. Equivalent number of transformed *ClearColi* cells were lysed and soluble and insoluble cells fractions were separated. All proteins were detected with anti-his antibody. Data are presented as mean ± STD of biological triplicate measurements.

## Data Availability

The datasets used and/or analyzed during the current study are available from the corresponding author on reasonable request.

## Abbreviations

IBs: Inclusion bodies
APP: Aggregation-prone peptide
iRFP: Near-infrared fluorescent protein
GFP: Green fluorescence protein
HS: Hot spot
HAS: Hot spot area
NHSA: Normalized HAS
a^4^vAHS: Average aggregation-propensity in each HS
AMP: Antimicrobial peptide
