# Supplementary material for: Title: insoluble proteins catch heterologous soluble proteins into inclusion bodies by intermolecular interaction of aggregating peptides
Source: Microb Cell Fact. 2021 Feb 2;20:30. doi: 10.1186/s12934-021-01524-3 (PMC7852131; doi:10.1186/s12934-021-01524-3)
Supplement: Supplementary file 1 — Additional file 1: Figure S1. Extended confocal microscopy visualization of E. coli ClearColi cells producing the recombinant fluorescent proteins. a Visual field of cells producing L6K2-containing constructs and VP1GFPH6 (aggregation control) analyzed in Fig. 2. b Visual fields of cells producing simultaneously GFP and EBFP2 constructs analyzed in Fig 5. Scale bar indicates 4 μm. [file 12934_2021_1524_MOESM1_ESM.docx]

**Carratalá et al**

**Figure S1. Extended confocal microscopy visualization of *E. coli ClearColi* cells producing the recombinant fluorescent proteins. a** Visual field of cells producing L6K2-containing constructs and VP1GFPH6 (aggregation control) analyzed in Fig. 2. **b** Visual fields of cells producing simultaneously GFP and EBFP2 constructs analyzed in fig 5. Scale bar indicates 4 μm.

**a**

**b**
