# Supplementary material for: Title: insoluble proteins catch heterologous soluble proteins into inclusion bodies by intermolecular interaction of aggregating peptides
Source: Microb Cell Fact. 2021 Feb 2;20:30. doi: 10.1186/s12934-021-01524-3 (PMC7852131; doi:10.1186/s12934-021-01524-3)
Supplement: Supplementary file 2 — Additional file 2: Figure S2. PEP-FOLD server-generated models of aggregation prone peptides fused to GFP scaffold protein. a Regular polypeptide helices in a right-handed alpha-helical conformation are shown. All structures are reproduced at the same scale. b Helical conformation of two L6K2-containing peptides (blue) in the presence of PT linker (grey). [file 12934_2021_1524_MOESM2_ESM.docx]

**Carratalá et al.**

**Figure S2. PEP-FOLD server-generated models of aggregation prone peptides fused to GFP scaffold protein. a.** Regular polypeptide helices in a right-handed alpha-helical conformation are shown. All structures are reproduced at the same scale. **b.** Helical conformation of two L6K2-containing peptides (blue) in the presence of PT linker (grey).

**a**


**b**
