# Supplementary material for: Title: insoluble proteins catch heterologous soluble proteins into inclusion bodies by intermolecular interaction of aggregating peptides
Source: Microb Cell Fact. 2021 Feb 2;20:30. doi: 10.1186/s12934-021-01524-3 (PMC7852131; doi:10.1186/s12934-021-01524-3)

**Carratalá et al.**

**Figure S3.** **DNA sequences of recombinant genes H6iRFPL6K2 and H6GFPL6K2 used in the study.** Translate tool from Expasy was used to obtain corresponding amino acid sequences. Clustalw was run to align amino acid sequences and Swiss Model to display 3D structures of the recombinant proteins.

> A7PL4_H6iRFPL6K2

Atgggccatcatcatcatcaccatgaaaacctgtattttcagggtatggcagaaggtagcgttgcacgtcagccggatctgctgacctgtgatgacgaaccgattcatattccgggtgcaattcagccgcatggtctgctgctggcactggcagcagatatgaccattgttgcaggtagcgataatctgccggaactgaccggtctggcaattggtgcactgattggtcgtagcgcagcagatgtttttgatagcgaaacccataatcgtctgaccattgcactggccgaaccgggtgcagcagtgggtgcaccgattaccgttggttttaccatgcgtaaagatgccggttttattggtagctggcatcgtcatgatcaactgatttttctggaactggaaccgcctcagcgtgatgttgcagaaccgcaggcattttttcgtcgtaccaatagcgcaattcgtcgtctgcaggcagcagaaaccctggaaagcgcatgtgcagcagcagcacaagaagttcgtaaaatcaccggttttgatcgcgtgatgatttatcgttttgcaagcgattttagcggtgaagttattgccgaagatcgttgtgcagaagttgaaagcaaactgggtctgcattatccggcaagcaccgttccggcacaggcacgtcgtctgtataccattaatccggttcgtattattccggacattaactatcgtccggttccggttacaccggatctgaatccggttaccggtcgtccgattgatctgagctttgcaattctgcgtagcgttagtccggttcatctggaatttatgcgtaatattggtatgcatggcaccatgagcattagcattctgcgtggtgaacgtctgtggggtctgattgtttgtcatcatcgtaccccgtattatgttgatctggatggtcgtcaggcatgtgaactggttgcacaggttctggcatggcagattggtgttatggaagaaccgacccctccgaccaccccgacaccgcctacaacaccgaccccgaccccattattactgctgctgctgaaaaaatga

>A6PL4_H6GFPL6K2

Atgcatcatcatcatcaccacgagaacctgtattttcagggtagcaaaggtgaagaactgtttaccggtgttgttccgattctggttgaactggatggtgatgttaatggccacaaattttcagttagcggtgaaggcgaaggtgatgcaacctatggtaaactgaccctgaaatttatctgtaccaccggcaaactgccggttccgtggccgaccctggttaccaccctgacctatggtgttcagtgttttagccgttatccggatcacatgaaacgccacgattttttcaaaagcgcaatgccggaaggttatgttcaagaacgtaccatctcctttaaagatgacggcaactataaaacccgtgccgaagttaaatttgaaggtgataccctggtgaatcgcattgaactgaaaggcatcgattttaaagaggatggtaatatcctgggccacaaactggaatataattataacagccacaacgtgtatatcaccgcagacaaacagaaaaatggcatcaaagccaacttcaaaatccgccataatattgaagatggtagcgtgcagctggcagatcattatcagcagaataccccgattggtgatggtccggttctgctgccggataatcattatctgagcacccagagcgcactgagcaaagatccgaatgaaaaacgtgatcacatggtgctgctggaatttgttaccgcagcaggtattacccatggtatggatgaactgtatccgacccctccgaccaccccgacaccgcctacaacaccgaccccgaccccattattactgctgctgctgaaaaaatga

<https://web.expasy.org/translate/>

(PT linker is shown in bold letters)

> H6iRFPL6K2

MGHHHHHHEN LYFQGMAEGS VARQPDLLTC DDEPIHIPGA IQPHGLLLAL AADMTIVAGS

DNLPELTGLA IGALIGRSAA DVFDSETHNR LTIALAEPGA AVGAPITVGF TMRKDAGFIG

SWHRHDQLIF LELEPPQRDV AEPQAFFRRT NSAIRRLQAA ETLESACAAA AQEVRKITGF

DRVMIYRFAS DFSGEVIAED RCAEVESKLG LHYPASTVPA QARRLYTINP VRIIPDINYR

PVPVTPDLNP VTGRPIDLSF AILRSVSPVH LEFMRNIGMH GTMSISILRG ERLWGLIVCH

HRTPYYVDLD GRQACELVAQ VLAWQIGVME E**PTPPTTPTP PTTPTPTP**LL LLLLKK

> H6GFPL6K2

MHHHHHHENL YFQGSKGEEL FTGVVPILVE LDGDVNGHKF SVSGEGEGDA TYGKLTLKFI

CTTGKLPVPW PTLVTTLTYG VQCFSRYPDH MKRHDFFKSA MPEGYVQERT ISFKDDGNYK

TRAEVKFEGD TLVNRIELKG IDFKEDGNIL GHKLEYNYNS HNVYITADKQ KNGIKANFKI

RHNIEDGSVQ LADHYQQNTP IGDGPVLLPD NHYLSTQSAL SKDPNEKRDH MVLLEFVTAA

GITHGMDELY **PTPPTTPTPP TTPTPTP**LLL LLLKK

<https://www.genome.jp/tools-bin/clustalw>

[clustalw.aln](https://www.genome.jp/tools-bin/pushfile?190531221524II3Qr+clustalw.aln)

CLUSTAL 2.1 multiple sequence alignment

H6iRFPL6K2 MGHHHHHHENLYFQGMAEGSVARQPDLLTCDDEPIHIPGAIQPHGLLLALAADMTIVAGS

H6GFPL6K2 -MHHHHHHENLYFQGSKG-----EELFTGVVPILVELDGDVNGHKFSVSGEGEGDATYG-

************* : : :.: * :: * : :: .: . *

H6iRFPL6K2 DNLPELTGLAIGALIGRSAADVFDSETHNRLTIALAEPGAAVGAPITVGFTMRKDAGFIG

H6GFPL6K2 ------------------------KLTLKFICTTGKLP---VPWPTLVTTLTYGVQCFSR

. * : : : * * * * *

H6iRFPL6K2 SWHRHDQLIFLELEPPQRDVAEPQAFFRRTNSAIRRLQAAETLESACAAAAQEVRKITGF

H6GFPL6K2 YPDHMKRHDFFKSAMPEGYVQERTISFK--DDGNYKTRAEVKFEGDTLVNRIELKGIDFK

.: .: *:: *: * * *: :.. : :* .:*. . *:: *

H6iRFPL6K2 DRVMIYRFASDFSGEVIAEDRCAEVESKLGLHYPASTVPAQARRLYTINPVRIIPDINYR

H6GFPL6K2 EDGNILGHKLEYN--------------------------------YNSHNVYITADKQKN

: * . ::. *. : * * .* : .

H6iRFPL6K2 PVPVTPDLNPVTGRPIDLSFAILRSVSPVHLEFMRNIGMHGTMSISILRGERLWGLIVCH

H6GFPL6K2 GIKANFKIRHNIEDGSVQLADHYQQNTPIGDGPVLLPDNHYLSTQSALSKDPNE------

: .. .:. :. :*: : . * : * * :

H6iRFPL6K2 HRTPYYVDLDGRQACELVAQVLAWQIGVMEEPTPPTTPTPPTTPTPTPLLLLLLKK

H6GFPL6K2 -------KRDHMVLLEFVTAAGITHGMDELYPTPPTTPTPPTTPTPTPLLLLLLKK

. * *:*: . : *************************

GFP 3D structure using Swiss Model


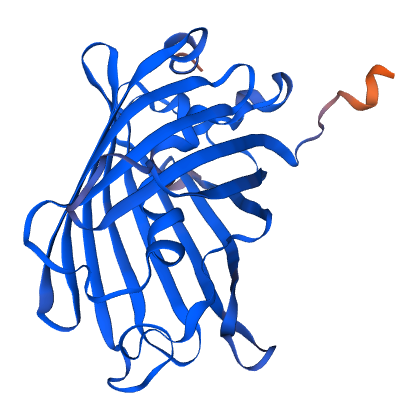


iRFP 3D structure using Swiss Model


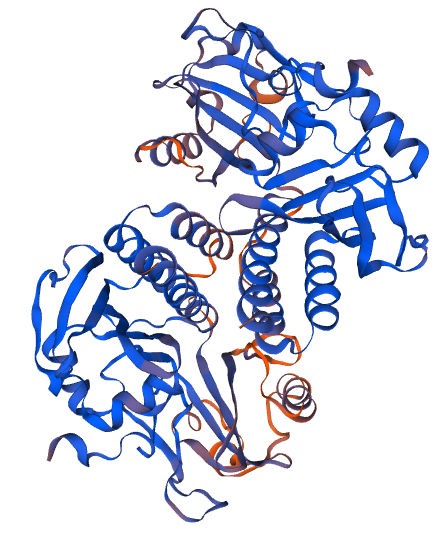

Supplement: Supplementary file 3 — Additional file 3: Figure S3. DNA sequences of recombinant genes H6iRFPL6K2 and H6GFPL6K2 used in the study. Translate tool from Expasy was used to obtain corresponding amino acid sequences. Clustalw was run to align amino acid sequences and Swiss Model to display 3D structures of the recombinant proteins. [file 12934_2021_1524_MOESM3_ESM.docx]
