# Supplementary material for: Title: insoluble proteins catch heterologous soluble proteins into inclusion bodies by intermolecular interaction of aggregating peptides
Source: Microb Cell Fact. 2021 Feb 2;20:30. doi: 10.1186/s12934-021-01524-3 (PMC7852131; doi:10.1186/s12934-021-01524-3)

**Carratalá et al.**

**Supplementary information**

**Figure S4.** **DNA sequence of recombinant gene VP1EBFP2H6 used in the study.** Translate tool from Expasy was used to obtain corresponding amino acid sequence. Clustalw was run to align amino acid sequence of GFP and EBFP2. Swiss Model was used to display 3D structure of EBFP2 protein.

>E4PL4-VP1EBFP2H6

ATGGAAACCACCACCACCGGTGAAAGCGCAGATCCGGTTACCACCACAGTTGAAAATTATGGTGGTGAAACCCAGGTTCAGCGTCGTCATCATACCGATGTTGCATTTGTTCTGGATCGTTTTGTTAAAGTTACCGTGAGCGATAATCAGCATACCCTGGATGTTATGCAGGCCCATAAAGATAATATTGTTGATGCACTGCTGCGTGCAGCAACCTATTATTTCAGCGATCTGGAAATTGCAGTTACCCATACCGGTAAACTGACCTGGGTTCCGAATGGTGCACCGGTTAGCGCACTGAATAATACCACCAATCCGACCGCATATCATAAAGGTCCGGTGACCCGTCTGGCACTGCCGTATACCGCACCGCATCGTGTTCTGGCAACCGCATATACCGGTACAACCACCTATACCGCAAGCGCACGTGGTGATCTGGCACATCTGACCACCACCCATGCACGTCATCTGCCGACCAGCTTTAACTTTGGTGCAGTTAAAGCAGAAACCATTACCGAACTGCTGGTTCGTATGAAACGTGCAGAACTGTATTGTCCGCGTCCGATTCTGCCGATTCAGCCGACCGGTGATCGTCATAAACAGCCGCTGGTTGCACCGGCAAAACAGCTGCTGGGTATTCCGAGCAAAGGTGAAGAACTGTTTACAGGTGTTGTGCCGATTCTGGTTGAACTGGATGGTGATGTTAATGGCCACAAATTTTCAGTTCGTGGTGAAGGCGAAGGTGATGCAACCAATGGCAAACTGACCCTGAAATTTATCTGTACCACAGGTAAACTGCCGGTTCCGTGGCCGACCCTGGTGACCACCCTGAGTCATGGTGTTCAGTGTTTTGCACGTTATCCGGATCACATGAAACAGCACGATTTTTTCAAAAGCGCAATGCCGGAAGGTTATGTTCAAGAACGTACCATCTTCTTCAAAGATGATGGCACCTATAAAACCCGTGCCGAAGTTAAATTTGAAGGTGATACCCTGGTTAACCGCATTGAACTGAAAGGTGTGGATTTTAAAGAGGATGGTAATATCCTGGGCCACAAACTGGAATATAATTTCAACAGCCACAACATCTATATCATGGCCGTGAAACAGAAAAACGGCATCAAAGTGAATTTCAAAATCCGCCATAATGTGGAAGATGGTTCAGTTCAGCTGGCAGATCATTATCAGCAGAATACCCCGATTGGTGATGGTCCGGTTCTGCTGCCGGATAGCCATTATCTGAGCACCCAGAGCGTTCTGAGCAAAGATCCGAATGAAAAACGTGATCACATGGTGCTGCTGGAATTTCGTACCGCAGCAGGTATTACCCTGGGTATGGATGAACTGTATAAACATCATCACCATCATCATTAA

<https://web.expasy.org/translate/>

METTTTGESA DPVTTTVENY GGETQVQRRH HTDVAFVLDR FVKVTVSDNQ HTLDVMQAHK

DNIVDALLRA ATYYFSDLEI AVTHTGKLTW VPNGAPVSAL NNTTNPTAYH KGPVTRLALP

YTAPHRVLAT AYTGTTTYTA SARGDLAHLT TTHARHLPTS FNFGAVKAET ITELLVRMKR

AELYCPRPIL PIQPTGDRHK QPLVAPAKQL LGIPSKGEEL FTGVVPILVE LDGDVNGHKF

SVRGEGEGDA TNGKLTLKFI CTTGKLPVPW PTLVTTLSHG VQCFARYPDH MKQHDFFKSA

MPEGYVQERT IFFKDDGTYK TRAEVKFEGD TLVNRIELKG VDFKEDGNIL GHKLEYNFNS

HNIYIMAVKQ KNGIKVNFKI RHNVEDGSVQ LADHYQQNTP IGDGPVLLPD SHYLSTQSVL

SKDPNEKRDH MVLLEFRTAA GITLGMDELY KHHHHHH

Orange: VP1 PDB: 1QGC_1

Blue: EBFP2

<https://www.genome.jp/tools-bin/clustalw>

[clustalw.aln](https://www.genome.jp/tools-bin/pushfile?190617154834vf3J4+clustalw.aln)

CLUSTAL 2.1 multiple sequence alignment

GFP ENLYFQGSKGEELFTGVVPILVELDGDVNGHKFSVSGEGEGDATYGKLTLKFICTTGKLP

EBFP -------SKGEELFTGVVPILVELDGDVNGHKFSVRGEGEGDATNGKLTLKFICTTGKLP

**************************** ******** ***************

GFP VPWPTLVTTLTYGVQCFSRYPDHMKRHDFFKSAMPEGYVQERTISFKDDGNYKTRAEVKF

EBFP VPWPTLVTTLSHGVQCFARYPDHMKQHDFFKSAMPEGYVQERTIFFKDDGTYKTRAEVKF

**********::*****:*******:****************** *****.*********

GFP EGDTLVNRIELKGIDFKEDGNILGHKLEYNYNSHNVYITADKQKNGIKANFKIRHNIEDG

EBFP EGDTLVNRIELKGVDFKEDGNILGHKLEYNFNSHNIYIMAVKQKNGIKVNFKIRHNVEDG

*************:****************:****:** * *******.*******:***

GFP SVQLADHYQQNTPIGDGPVLLPDNHYLSTQSALSKDPNEKRDHMVLLEFVTAAGITHGMD

EBFP SVQLADHYQQNTPIGDGPVLLPDSHYLSTQSVLSKDPNEKRDHMVLLEFRTAAGITLGMD

***********************.*******.***************** ****** ***

GFP ELY

EBFP ELY

***

EBFP 3D structure using Swiss Model


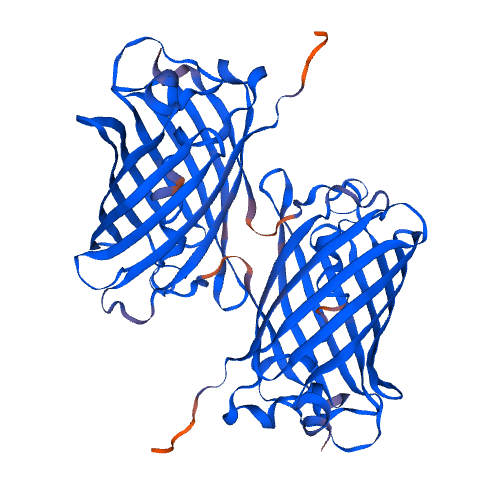

Supplement: Supplementary file 4 — Additional file 4: Figure S4. DNA sequence of recombinant gene VP1EBFP2H6 used in the study. Translate tool from Expasy was used to obtain corresponding amino acid sequence. Clustalw was run to align amino acid sequence of GFP and EBFP2. Swiss Model was used to display 3D structure of EBFP2 protein. [file 12934_2021_1524_MOESM4_ESM.docx]
