# Supplementary material for: Title: insoluble proteins catch heterologous soluble proteins into inclusion bodies by intermolecular interaction of aggregating peptides
Source: Microb Cell Fact. 2021 Feb 2;20:30. doi: 10.1186/s12934-021-01524-3 (PMC7852131; doi:10.1186/s12934-021-01524-3)
Supplement: Supplementary file 5 — Additional file 5: Figure S5. Relative solubility (%) of recombinant proteins. Detection of H6GFPL6K2, VP1GFPH6 and VP1EBFP2H6 in the soluble and insoluble cell fractions of ClearColi analyzed by Western Blotting. Equivalent number of transformed ClearColi cells were lysed and soluble and insoluble cells fractions were separated. All proteins were detected with anti-his antibody. Data are presented as mean ± STD of biological triplicate measurements. [file 12934_2021_1524_MOESM5_ESM.docx]

**Carratalá et al.**

**Figure S5.** **Relative solubility (%) of recombinant** **proteins.** Detection of H6GFPL6K2, VP1GFPH6 and VP1EBFP2H6 in the soluble and insoluble cell fractions of *ClearColi* analyzed by Western Blotting. Equivalent number of transformed *ClearColi* cells were lysed and soluble and insoluble cells fractions were separated. All proteins were detected with anti-his antibody. Data are presented as mean ± STD of biological triplicate measurements.
